# A national-scale dataset of arable plant abundance from citizen science surveys of swedish field margins

**DOI:** 10.1016/j.dib.2026.112602

**Published:** 2026-02-16

**Authors:** Rebecca C. Örnberg, Alexander Menegat, Darwin T. Hickman, Alistair G. Auffret, Johan Nilsson, Gunnar Nyborg, Sofie Wikberg, Jan Y. Andersson, Sebastian Sundberg

**Affiliations:** a Swedish University of Agricultural Sciences, Faculty of Natural Resources and Agricultural Sciences, Department of Crop Production Ecology, Uppsala 756 51, Sweden; b Uppsala University, Biology Education Centre, Uppsala 752 36, Sweden; c Swedish Species Information Centre, Swedish University of Agricultural Sciences, PO Box 7007, Uppsala SE-750 07, Sweden; d Division of IT, Swedish University of Agricultural Sciences, Uppsala SE-750 07, Sweden; e Swedish Botanical Society, Kungsängens gård 206, Uppsala SE-753 23, Sweden; f Swedish University of Agricultural Sciences, Faculty of Natural Resources and Agricultural Sciences, Department of Ecology, Uppsala 756 51, Sweden

**Keywords:** Biodiversity, Agriculture, Agricultural weed, Plant conservation, Plant community

## Abstract

We present here a first-of-its-kind survey of field margin flora in Sweden. The survey was carried out in summer 2020 – 2023, covering most of Sweden’s major agricultural regions. Volunteer botanists surveyed a 100 × 1 m transect at the edge of the crop, estimating abundances of all non-crop plants growing there. We later cleaned the data by cross-referencing surveyor comments and reports with data on management and soil, and filtered it to only include herbaceous plants growing in the field at the height of the growing season, before harvest. In total, 7364 observations from 442 species found in 294 sites, ranging from Skåne in the south to Västerbotten in the north, were retained in the data. These data offer a unique insight into the present state of Swedish field margin plant communities, from a fine to a large scale. They are useful for community studies, and can aid in making informed decisions on management and conservation of plants growing on, or near, agricultural land.

Specifications Table

**Table:** 

Subject	Biology
Specific subject area	Large-scale botanical survey of field margins in Swedish agroecosystems
Type of data	Table (Excel file, csv file, txt file). Filtered.
Data collection	The data were collected as part of a national citizen science survey project engaging volunteer botanists. Surveys were carried out at the edge of the crop field along 100 × 1 m transects, in early summer to early autumn. All field types except pasture and long-term ley were considered, and all vascular plants were recorded. The dataset was cleaned and filtered with the help of surveyors’ comments, and databases with field crop and soil information. The resulting data file contains 7364 entries of 442 species, or lower taxa/species complexes from 294 sites.
Data source location	Country: Sweden
Data accessibility	Repository name: Swedish National Data Service (SND) Data identification number: https://doi.org/10.5878/xnzn-7k56 Direct URL to data: https://researchdata.se/sv/catalogue/dataset/2025–338/1 Raw data can be accessed through the Swedish Species Observation System (*Artportalen*) by the URL https://www.artportalen.se/ViewSighting/SharedSearch?storedSearchId=24839&identifier=F1643741 or by searching for the project “*Åkerogräsinventering*” at https://www.artportalen.se.
Related research article	None

## 1 Value of the Data

• These data are from a first-of-its-kind, large-scale survey of arable plants in Sweden, providing a unique perspective on the plant assemblages, occurrences, and distributions in Swedish agricultural field margins.
• The dataset has been collected in consideration of a wide range of applications, and is useful for both large- and fine-scale studies that require extensive plant abundance and diversity data.
• This dataset is useful for a wide range of people, from researchers in ecology and agronomy to decision makers, governmental bodies and farmers interested in biodiversity conservation, plant distribution, and plant community composition from a fine to a large scale.

## 2 Background

In this article is presented data from a nation-wide botanical survey of Swedish field margins. Here we describe the cleaned and filtered dataset for easy use in analyses [1].

The survey was organised by the Swedish Botanical Society (SBF), the Swedish University of Agricultural Sciences (SLU) Species Information Centre (*Artdatabanken*), and the Departments of Ecology and Crop Production Ecology at SLU, the years 2020 to 2023. It engaged volunteer botanists in taking inventory of vascular plants growing in field margins of agricultural land across Sweden. The project was available for participation for members of the Swedish Botanical Society (SBF), and participants could report interest and access instructions *via* SBF or SLU *Artdatabanken*, making this one of the larger citizen science projects, and the first nationwide inventory of arable plants in Sweden to date. Throughout the survey period, awareness was raised and updates provided through publications in national botanical magazines, regular e-mails and newsletters to participants [[2], [3], [4], [5]].

## 3 Data Description

The data file is divided into two sheets [1]. The first sheet contains a brief description of the data and its recommended usage. The second sheet contains the species data, which is the focus of this article. Available for download are also a .csv file and a .txt file with the survey data, and two README files in English (README_EN) and Swedish (README_SV) with a shortened description of the contents, as well as a cleaning log with information of how data has been cleaned and filtered.

**Species data:**

The species datasheet contains a total of 7364 species observations of 442 species, subspecies and species complexes from 294 sites. Each row is an observation, and each column describes an aspect of the site or the species. The columns are as follows:

1. **Taxon ID:** The Taxon ID is connected to the Dyntaxa taxonomic database at the Species Information Centre, managed by SLU. Each species, subspecies or species complex is uniquely identifiable by this code. The code can be used as an alternative to species name on the website artfakta.se to find information about the species, its habitat, taxonomy and more.
2. **Scientific Name:** This column contains the scientific name of the species. Note that there are species included that are either crop species, or are commonly sown as cover or ley crops.
3. **Abundance:** Values in this column give the number of plants found within the surveyed transect. These were typically estimates, rather than absolute abundance, especially for higher values.
4. **Unit:** Gives the counted unit of the surveyed plants (plants/tufts or stalks/stems/shoots), when this was provided by the surveyor.
5. **Site Number:** This column describes the site where the plant was found. Note that Site 279 was surveyed twice, in 2022 (279_1) and 2023 (279_2).
6. **Coordinates:** The coordinates give the approximate mid-point of the surveyed transect. They are given in WGS 84 projection at a resolution of 50 m to include the whole 100 m transect.
7. **County, municipality, province and parish:** The columns “county” and “municipality” describe in which of Sweden’s 21 administrative larger and 290 smaller units the plant was found, respectively. Provinces largely overlap with counties, while parishes are more fine scale than municipality (there are around 2350 parishes in Sweden). These have a historical and cultural usage, and may be useful in comparisons over long timescales since the provincial and parish borders do not change over time, unlike county borders.
8. **Date:** Gives the date the survey was conducted.
9. **Year:** Gives the year the survey was conducted.
10. **Unclear Identification:** Binary classification with two levels. False indicates the surveyor was confident in the identification, while true indicates they could not confidently identify the species, lower taxa, or species complex.
11. **Conservation Status:** The Red List status of the plant in Sweden at the time of survey (according to the 2020 Swedish Red List [6]), as evaluated according to the criteria and categories in IUCN guidelines at regional and national level [7], which gives an estimate of the conservation status and threat to a given species. If a subspecies has not been evaluated, it will inherit the conservation status of the species. Aggregates and sections may include taxa with diverse Red List classes.
12. **Site-type:** Gives whether the site was in the priority grid or free-choice (see Experimental Design, Materials and Methods, sub-header “Survey instruction” for more information).
13. **Management:** Shows whether the field was under organic or conventional management. A total of 107 organic and 187 conventional fields were included.
14. **Crop:** Describes which crop was growing in the field at the time of survey.
15. **Time of Sowing:** Gives whether the grown crop is a spring or autumn crop, for the crops where such a distinction is relevant and known.
16. **SGU Soil Type and Soil Type Correction:** Describes the soil type of the field, as indicated by the Swedish Geological Survey (SGU). In some cases, a correction of the soil type was necessary because an unlikely soil type had been registered (*e.g.* water or primary rock). This is done in the column “Soil Type Correction”.
17. **Field Size:** Gives the size of the field in hectares (see Fig. 1 for distribution across the dataset).

**Fig. 1:**
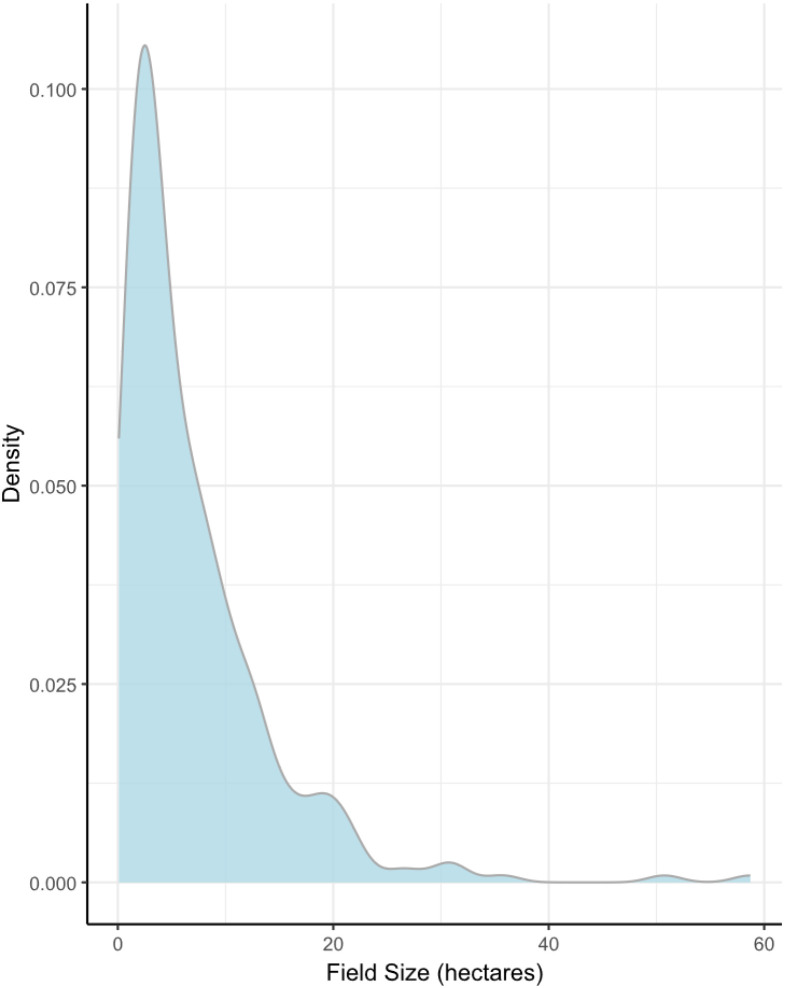
Size distribution of surveyed fields. The density curve describes the spread of probabilities of different field sizes. More values occur around where the curve peaks, *i.e.* most fields in the data are between 1 and 10 hectares.
18. **Field Entrances:** Indicates how many field entrances were along the surveyed transect.
19. **Field Corners:** Indicates how many field corners were in the surveyed transect.

## 4 Experimental Design, Materials and Methods

### 4.1 Survey material

• A phone or GPS and camera to record coordinates and take photos
• Hand lens
• Equipment to measure the width (1 m) and length (100 m) of the transect
• Material to press and preserve plant material
• Plant identification literature and keys
• Survey protocol
• Map of the area

### 4.2 Survey instructions

**Fig. 2:**
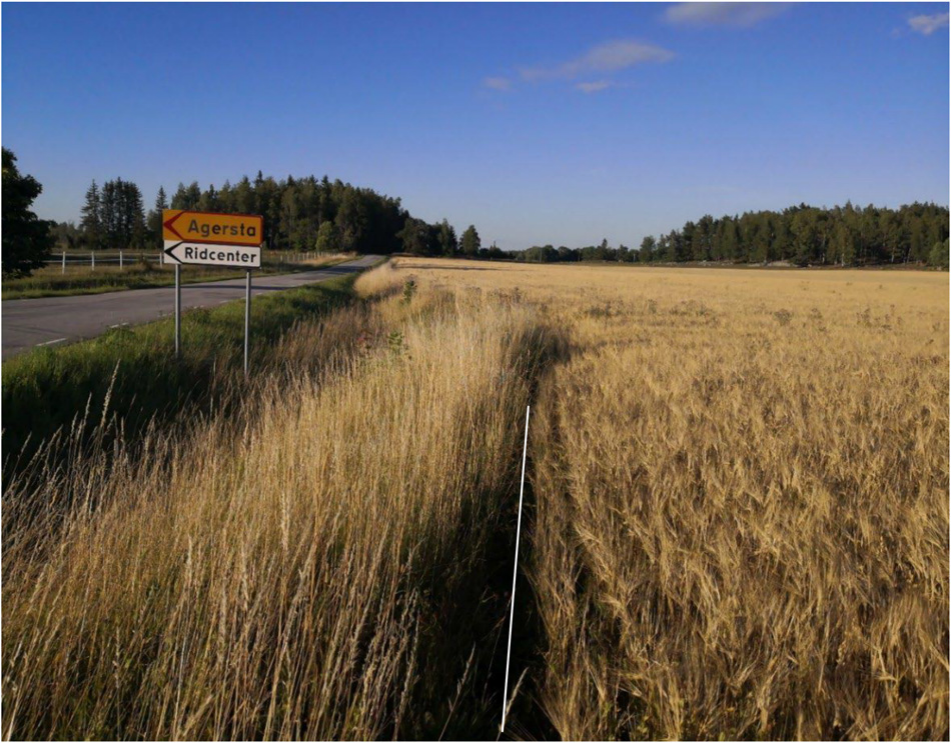
Photo of the border between the cropped and non-cropped parts of a field. Only what was to the right of the white line was surveyed.

Fields: All field types were of interest. This included fields with annual or perennial crops or fallow fields, both organic and conventional. Pastureland and fields with permanent grass were excluded from the survey. A 100 × 1 m transect was surveyed along each field margin (fields with a circumference of <100 m were excluded, see Fig. 2 for an example of the surveyed region). Only tilled/harrowed soil was included in the transect. Surveyors partaking in the project could choose fields in two ways, selecting a field of their own, or one within the priority grid. The priority grid consisted of 300 2 × 2 km squares systematically placed every 25 km across arable regions of Sweden. The priority grid was established to facilitate long term monitoring and comparison, as well as correction of bias in free-choice fields. A field fully or partially within such a square was marked a “priority field”. If the surveyor chose to freely select a field, they were encouraged to avoid already-inventoried fields, and not to select fields based on expected interesting observations. Surveyors were also encouraged to survey both organically and conventionally managed fields, with a variety of crops, to cover as much of the current agricultural landscape as possible.

**Plants:** All vascular plants found on the 100 m long transect were recorded, if they were rooted within the 1 m breadth of the transect. Surveyors were asked to identify plants to as much detail as they could provide, from genus and aggregate levels to subspecies and varieties, especially for characteristic arable plants. For taxa that were rare or difficult to identify, surveyors were also asked to collect material for validation and documentation, if that species was not protected. For the protected species *Anthemis cotula, Galeopsis angustifolia* and *Sagina apetala*, permission to collect material was given by the country administrations boards of Skåne, Blekinge, Gotland, Kalmar, Halland, Västra Götaland and Östergötland. No protected species were recorded, however. Plants were counted as number of individuals or tufts, except for grasses and plants growing with runners or in large stands (*e.g. Lathyrus*), where number of strands, stems or shoots were counted. Abundances were reported using logarithmically increasing abundance classes, with mid-points used as representative values, with some exceptions for rare or scarce plants.

**Time of survey:** The surveys were recommended to be carried out before crop harvest, from the middle of June to beginning of August. As the growing season starts earlier and ends later in the south compared to the north of Sweden, some flexibility was allowed to account for this variability. Time spent surveying the field was left to the discretion of the surveyor, with emphasis being on recording all taxa present in the transect.

**Reporting:** All reporting of species was done through the Species Observation System (https://www.artportalen.se/), in the specific project “*Åkerogräsinventering*”, managed by SLU.

### 4.3 Data cleaning and error-correction

To ensure its consistency, the data have been thoroughly checked and harmonised. A primary cleaning was done to reduce errors by correcting coordinates in GIS to ensure they were at the edge of the field. Correction of coordinates, crop data, and management was done in communication with the surveyors. In a second cleaning we checked each site for inconsistencies and discrepancies. Comments by the surveyors, and cross-referencing with available soil and agricultural data through SGU and the Swedish Board of Agriculture (“*Jordbruksverket”)*, ensured that confidence could be placed in retained data entries. All entries that could not be confirmed with certainty were removed from the present dataset. Some reasons for the removal of a site included:

• Multiple visits had been made without clear indication what was counted or when
• Plant species had only been noted and not quantified
• There were discrepancies in crop data
• The surveyor did not follow the instructions for the survey
• The field was surveyed after harvest

A total of 72 sites were removed due to these inconsistencies. Note that we have chosen a conservative approach to the cleaning, electing to remove sites that could not be confirmed with certainty by cross-validation with available databases. In many of these cases, the surveyor reports are likely to be correct, though they could not be validated. A full description of what was removed and why can be found in the cleaning log in Supplementary Materials S1, or for download at the data repository [1].

## Limitations

As always with citizen science projects, there is a risk of bias towards more “interesting” locations. While our cleaning efforts have reduced the margin of error, it can never be completely eradicated. Users should be aware that there is a risk of unexpected noise in the data due to its size and nature, and act accordingly. Additionally, while there is a spread of locations, with representation from all major agricultural areas of Sweden, there is a slight skew towards fields in central-eastern Sweden.

## Ethics Statement

The authors hereby confirm that they have read and follow the ethical requirements for publication in Data in Brief, and that the presented work does not include human subjects, animal experiments or data collected from social media platforms.

## CRediT Author Statement

**Rebecca C. Örnberg:** Validation, Data Curation, Writing Original Draft, Visualisation; **Alexander Menegat:** Conceptualisation, Methodology, Writing - Review & Editing, Supervision; **Darwin T. Hickman:** Writing - Review & Editing, Supervision; **Alistair G. Auffret:** Conceptualisation, Methodology, Writing - Review & Editing; **Johan Nilsson:** Conceptualisation, Methodology, Writing - Review & Editing, Data Curation; **Gunnar Nyborg:** Conceptualisation, Methodology, Writing - Review & Editing, Data Curation; **Sofie Wikberg:** Conceptualisation, Methodology, Writing - Review & Editing, Data Curation; **Jan Y. Andersson:** Conceptualisation, Methodology, Writing - Review & Editing, Data Curation; **Sebastian Sundberg:** Conceptualisation, Methodology, Investigation, Resources, Data Curation, Writing - Review & Editing, Supervision, Project Administration & Funding acquisition.

## Data Availability

(Swedish National Data Service (SND)).Data for: A National-scale Dataset or Arable Plant Abundance from Citizen Science Surveys of Swedish Field Margins (Original data)

## Appendix Supplementary materials

Image, application 1
