# Supplementary material for: A national-scale dataset of arable plant abundance from citizen science surveys of swedish field margins
Source: Data Brief. 2026 Feb 16;65:112602. doi: 10.1016/j.dib.2026.112602 (PMC12950477; doi:10.1016/j.dib.2026.112602)
Supplement: Supplementary file 1 [file mmc1.docx]

Supplementary Material: Cleaning log

Sites removed:

NOTE: In the removal of sites, we have elected to be very conservative and excluded all sites that could not be validated with certainty. In some, if not most, of these cases, the data reported by the surveyor are likely to be correct. However, to ensure absolute confidence in the reported data, they were excluded from the cleaned dataset. Should the user wish to include the data from these sites from the raw data, there is a detailed account of all sites that were removed and the reason for that removal below. The site number correspond to the column *“Intern lokalnumrering”* (internal site numbering) in the raw data.

1. Site 86
   1. All entries only present, comment says they were too small to be properly identified.
2. Site 45
   1. 2 visits 5 days apart without explanation, some plants were repeated.
3. Site 79
   1. 2 visits 1 month apart without explanation, some plants were repeated.
4. Site 341
   1. Repeated plants, 2 observations on the same day.
5. Site 20
   1. Quantification missing for some observations.
6. Site 250
   1. Quantification missing for some observations.
7. Site 52
   1. . Quantification missing for some observations.
8. Site 280
   1. Quantification missing for some observations.
9. Site 202
   1. Quantification missing for some observations.
   2. No Block-ID for field, field data could not be confirmed
10. Site 113
    1. All or some plants had not been counted without explanation. Quantification missing for some observations.
11. Site 114
    1. Quantification missing for some observations.
    2. Plants outside transect had been included.
12. Site 129
    1. Quantification missing for some observations.
13. Site 345
    1. Quantification missing for some observations.
14. Site 144
    1. Quantification missing for some observations.
15. Site 234
    1. Quantification missing for some observations.
16. Site 155
    1. Quantification missing for some observations,
17. Site 161
    1. Quantification missing for some observations.
18. Site 162
    1. Quantification missing for some observations.
19. Site 26
    1. Coverage instead of # individuals for some entries
20. Site 132
    1. Coverage instead of # individuals for some entries.
21. Site 78
    1. Visited twice within a year
22. Site 349
    1. Multiple visits, unclear how quantification had been done.
23. Site 223
    1. Visited twice in same year
24. Site 41
    1. Surveyed after harvest
25. Site 300
    1. Surveyed after harvest
26. Site 87
    1. Surveyed after harvest
27. Site 110
    1. Surveyed after harvest
28. Site 111
    1. Surveyed after harvest
29. Site 130
    1. Surveyed straight through field instead of edge transect.
30. Site 165
    1. Missing block-ID, field data could not be confirmed.
31. Site 180
    1. Missing block-ID, field data could not be confirmed.
32. Site 181
    1. Missing block-ID, field data could not be confirmed.
33. Site 260
    1. Missing block-ID, field data could not be confirmed.
34. Site 321
    1. Missing block-ID, field data could not be confirmed.
35. Site 342
    1. Missing block-ID, field data could not be confirmed.
36. Site 159
    1. Missing block-ID, field data could not be confirmed.
37. Site 35_1, 35_2
    1. Discrepancy between what crop was reported by the field surveyor of arable plants and what was recorded in the crop database at the Swedish Board of Agriculture.
38. Site 55
    1. Discrepancy between what crop was reported by the field surveyor of arable plants and what was recorded in the crop database at the Swedish Board of Agriculture.
39. Site 95
    1. Discrepancy between what crop was reported by the field surveyor of arable plants and what was recorded in the crop database at the Swedish Board of Agriculture.
40. Site 204
    1. Discrepancy between what crop was reported by the field surveyor of arable plants and what was recorded in the crop database at the Swedish Board of Agriculture.
41. Site 216
    1. Discrepancy between what crop was reported by the field surveyor of arable plants and what was recorded in the crop database at the Swedish Board of Agriculture.
42. Site 247
    1. Discrepancy between what crop was reported by the field surveyor of arable plants and what was recorded in the crop database at the Swedish Board of Agriculture.
43. Site 353
    1. Discrepancy between what crop was reported by the field surveyor of arable plants and what was recorded in the crop database at the Swedish Board of Agriculture.
44. Site 5
    1. Discrepancy between what crop was reported by the field surveyor of arable plants and what was recorded in the crop database at the Swedish Board of Agriculture.
45. Site 19
    1. Discrepancy between what crop was reported by the field surveyor of arable plants and what was recorded in the crop database at the Swedish Board of Agriculture.
46. Site 68
    1. Discrepancy between what crop was reported by the field surveyor of arable plants and what was recorded in the crop database at the Swedish Board of Agriculture.
47. Site 70
    1. Discrepancy between what crop was reported by the field surveyor of arable plants and what was recorded in the crop database at the Swedish Board of Agriculture.
48. Site 92
    1. Discrepancy between what crop was reported by the field surveyor of arable plants and what was recorded in the crop database at the Swedish Board of Agriculture.
49. Site 152
    1. Discrepancy between what crop was reported by the field surveyor of arable plants and what was recorded in the crop database at the Swedish Board of Agriculture.
50. Site 178
    1. Discrepancy between what crop was reported by the field surveyor of arable plants and what was recorded in the crop database at the Swedish Board of Agriculture.
51. Site 188
    1. Discrepancy between what crop was reported by the field surveyor of arable plants and what was recorded in the crop database at the Swedish Board of Agriculture.
52. Site 190
    1. Discrepancy between what crop was reported by the field surveyor of arable plants and what was recorded in the crop database at the Swedish Board of Agriculture.
53. Site 203
    1. Discrepancy between what crop was reported by the field surveyor of arable plants and what was recorded in the crop database at the Swedish Board of Agriculture.
54. Site 206
    1. Discrepancy between what crop was reported by the field surveyor of arable plants and what was recorded in the crop database at the Swedish Board of Agriculture.
55. Sites 226 & 359 (same site)
    1. Discrepancy between what crop was reported by the field surveyor of arable plants and what was recorded in the crop database at the Swedish Board of Agriculture.
56. Site 242
    1. Discrepancy between what crop was reported by the field surveyor of arable plants and what was recorded in the crop database at the Swedish Board of Agriculture.
57. Site 243
    1. Discrepancy between what crop was reported by the field surveyor of arable plants and what was recorded in the crop database at the Swedish Board of Agriculture.
58. Site 257
    1. Discrepancy between what crop was reported by the field surveyor of arable plants and what was recorded in the crop database at the Swedish Board of Agriculture.
59. Site 271
    1. Discrepancy between what crop was reported by the field surveyor of arable plants and what was recorded in the crop database at the Swedish Board of Agriculture.
60. Site 287
    1. Discrepancy between what crop was reported by the field surveyor of arable plants and what was recorded in the crop database at the Swedish Board of Agriculture.
61. Site 365
    1. Discrepancy between what crop was reported by the field surveyor of arable plants and what was recorded in the crop database at the Swedish Board of Agriculture.
62. Site 246
    1. Surveyed after harvest
63. Site 248
    1. Discrepancy between what crop was reported by the field surveyor of arable plants and what was recorded in the crop database at the Swedish Board of Agriculture.
64. Site 183
    1. Discrepancy between what crop was reported by the field surveyor of arable plants and what was recorded in the crop database at the Swedish Board of Agriculture.
65. Site 307
    1. Surveyed after harvest
66. Site 323
    1. Surveyed after harvest
67. Site 118
    1. Discrepancy between what crop was reported by the field surveyor of arable plants and what was recorded in the crop database at the Swedish Board of Agriculture.
68. Site 339
    1. Surveyed after harvest
69. Site 147
    1. Surveyed after harvest
70. Site 363
    1. Surveyed after harvest
71. Site 320
    1. Most likely a flowering strip that had been surveyed
72. Site 269
    1. Discrepancy between what crop was reported by the field surveyor of arable plants and what was recorded in the crop database at the Swedish Board of Agriculture.

Crops in brackets:

These entries are included in the raw data, and describe the currently growing crop in some of the fields. They have been excluded from the clean dataset.

1. × *Triticosecale rimpaui*
2. *Avena sativa*
3. *Brassica napus* subsp. *Napus*
4. *Fagopyrum esculentum*
5. *Hordeum vulgare* (including varieties var. *distichon* and var. *vulgare*)
6. *Linum usitatissimum*
7. *Lupinus angustifolius*
8. *Phaseolus vulgaris*
9. *Phleum pratense*
10. *Pisum sativum* var. *arvense*
11. *Pisum sativum*
12. *Poa pretensis*
13. *Raphanus sativus* var. o*leiformis*
14. *Secale cereale*
15. *Solanum tuberosum*
16. *Trifolium pratense* (var. s*ativum*)
17. *Triticum aestivum*
18. *Vicia faba*
19. *Zea mays*

Other species removed:

These species were removed either because they were growing in the field where the same species had been sown, or because it was considered that they function on a different scale than forbs (treed and woody plants).

1. *Solanum tuberosum* from 400 m S Svälte vid avf Ramsvik
   1. Removed because there was a potato patch within the field
2. *Phleum pratense* from S om Pal-Lars, Långhed Alfta
   1. Removed because it has not been counted – likely to be sown.
3. *Acer platanoides*
   1. Tree
4. *Corylus avellana*
   1. Tree
5. *Fraxinus excelsior*
   1. Tree
6. *Pinus sylvestris*
   1. Tree
7. *Populus tremula*
   1. Tree
8. *Quercus robur*
   1. Tree
9. *Sorbus aucuparia*
   1. Tree
10. *Prunus padus*
    1. Tree/woody plant
11. *Salix caprea*
    1. Tree/woody plant
12. *Prunus spinosa*
    1. Woody plant
13. *Symphoricarpos albus*
    1. Woody plant
14. *Rosa* (+ *Rosa canina* agg. and *Rosa vosagiaca*)
    1. Woody shrubs
15. *Rubus caesius*
    1. Shrub species
16. *Rubus gothicus*
    1. Shrub species
17. *Rubus idaeus*
    1. Shrub species
18. *Rubus plicatus*
    1. Shrub species
19. *Malus sylvestris*
    1. Woody plant
20. *Picea abies*
    1. Tree/woody species
21. Alnus incana
    1. Tree/woody species
22. Crataegus rhipidophylla
    1. Tree/woody species

Potential crop volunteers / often sown species in Sweden (found in the dataset):

These species are included in the dataset when they are found in fields where they had not been sown in the present year. Note that all of these are commonly sown, and some do not reproduce on their own in Sweden.

1. *Avena sativa*
   1. Non-reproducing in the wild
2. *Brassica napus* subsp. *napus*
   1. Non-reproducing in the wild
3. *Brassica oleracea*
   1. Non-reproducing in the wild
4. *Brassica rapa* subsp. *oleifera*
   1. Non-reproducing in the wild
5. *Cichorium intybus*
6. *Dactylis glomerata*
7. *Daucus carota*
   1. Exists as both a cultivated form and a wild form (subsp. *carota)*.
8. *Fagopyrum esculentum*
   1. Non-reproducing in the wild
9. *Festuca rubra*
10. *Helianthus annuus*
    1. Non-reproducing in the wild
11. *Hordeum vulgare, Hordeum vulgare* var. *distichon*
    1. Non-reproducing in the wild
12. *Lepidium campestre*
13. *Linum usitatissimum*
    1. Non-reproducing in the wild
14. *Lolium multiflorum*
    1. Non-native but reproducing
15. *Lolium perenne*
16. *Lotus corniculatus*
17. *Medicago sativa* subsp. *sativa*
18. *Pastinaca sativa*
19. *Phacelia tanacetifolia*
    1. Non-reproducing in Sweden
20. *Phaseolus vulgaris*
    1. Non-reproducing in the wild
21. *Phleum pratense*
22. *Pisum sativum, Pisum sativum* var. *arvense*
    1. Non-reproducing in the wild
23. *Poa pratensis*
24. *Raphanus sativus*
    1. Non-reproducing in Sweden
25. *Secale cereale*
    1. Non-reproducing in the wild
26. *Schedonorus arundinaceus*
27. *Schedonorus pratensis*
28. *Trifolium hybridum*
29. *Trifolium incarnatum*
    1. Non-reproducing in Sweden
30. *Trifolium pratense*
31. *Trifolium repens*
32. *Trifolium resupinatum*
    1. Non-reproducing in Sweden
33. *Triticosecale* (*Triticosecale rimpaui*)
    1. Non-reproducing in the wild
34. *Triticum aestivum*
    1. Non-reproducing in the wild
35. *Vicia villosa*

Crop translations (Swedish 🡪 English):

Blandsäd 🡪 Grain mix

Blålupin 🡪 Narrowleaf lupin

Bovete 🡪 Buckwheat

Böna 🡪 Field beans

Havre 🡪 Oats

Gotlandslins 🡪 Gotland lentil

Houngsfacelia 🡪 Lacy phacelia

Korn 🡪 Barley

Rödklöver 🡪 Red clover

Ärt 🡪 Pea

Fodervicker 🡪 Common vetch

Lin 🡪 Flax

Majs 🡪 Maize

Oljerättika 🡪 Oilseed radish

Raps 🡪 Rapeseed

Råg 🡪 Rye

Rågvete 🡪 Triticale

Sockerbeta 🡪 Sugar beet

Vete 🡪 Wheat

Klöver 🡪 Clover

Soil type translations (Swedish 🡪 English):

Berg 🡪 Rock

Glacial growsilt – finsand 🡪 Glacial coarse silt to fine sand

Glacial lera 🡪 Glacial clay

Glacial silt 🡪 Glacial silt

Gyttja 🡪 Gyttja

Gyttjelera (eller lergyttja) 🡪 Gyttja clay (or clay gyttja)

Isälvssediment 🡪 Glaciofluvial sediment

Isälvssediment, grus 🡪 Glaciofluvial gravel

Isälvssediment, sant 🡪 Glaciofluvial sand

Kärrtorv 🡪 Fen peat

Lera 🡪 Clay

Lera—silt 🡪 Clay to silt

Lerig morän 🡪 Till, clay content 5-15%

Morän 🡪 Till

Moränfinlera 🡪 Clay till, clay content >25%

Morängrovlera 🡪 Clay till, clay content 15-25%

Moränlera eller lerig morän 🡪 Clay till or clayey till

Postglacial finlera 🡪 Postglacial clay, clay content >25%

Postglacial finsand 🡪 Postglacial fine sand

Postglacial grovlera 🡪 Postglacial clay, clay content 15-25%

Postglacial lera 🡪 Postglacial clay

Postglacial sand 🡪 Postglacial sand

Postglacial sand—grus 🡪 Postglacial sand to gravel

Postglacial silt 🡪 Postglacial silt

Sandig morän 🡪 Sandy till

Sedimentärt berg 🡪 Sedimentary rock

Silt 🡪 Silt

Svallsediment, grus 🡪 Wave-washed gravel

Svämsediment, ler—silt 🡪 Young fluvial sediment, clay to silt

Svämsediment, sand 🡪 Young fluvial sediment, sand

Torv 🡪 Peat

Urberg 🡪 Crystalline rock

Älvsediment, grosilt—finsand

Älvsediment, sand 🡪 Fluvial sediment, sand
